# Endogenous Mechanisms of Craniomaxillofacial Repair: Toward Novel Regenerative Therapies

**DOI:** 10.3389/froh.2021.676258

**Published:** 2021-05-12

**Authors:** Heather E. desJardins-Park, Shamik Mascharak, Michael T. Longaker, Derrick C. Wan

**Affiliations:** ^1^Division of Plastic and Reconstructive Surgery, Hagey Laboratory for Pediatric Regenerative Medicine, Stanford School of Medicine, Department of Surgery, Stanford, CA, United States; ^2^Institute for Stem Cell Biology and Regenerative Medicine, Stanford School of Medicine, Stanford, CA, United States

**Keywords:** regeneration, scarring, wound healing, fibrosis, oral mucosa, skull, facial bones, teeth

## Abstract

In the fields of oral and craniomaxillofacial surgery, regeneration of multiple tissue types—including bone, skin, teeth, and mucosal soft tissue—is often a desired outcome. However, limited endogenous capacity for regeneration, as well as predisposition of many tissues to fibrotic healing, may prevent recovery of normal form and function for patients. Recent basic science research has advanced our understanding of molecular and cellular pathways of repair in the oral/craniofacial region and how these are influenced by local microenvironment and embryonic origin. Here, we review the current state of knowledge in oral and craniomaxillofacial tissue repair/regeneration in four key areas: bone (in the context of calvarial defects and mandibular regeneration during distraction osteogenesis); skin (in the context of cleft lip/palate surgery); oral mucosa (in the context of minimally scarring repair of mucosal injuries); and teeth (in the context of dental disease/decay). These represent four distinct healing processes and outcomes. We will discuss both divergent and conserved pathways of repair in these contexts, with an eye toward fundamental mechanisms of regeneration vs. fibrosis as well as translational research directions. Ultimately, this knowledge can be leveraged to develop new cell-based and molecular treatment strategies to encourage bone and soft tissue regeneration in oral and craniomaxillofacial surgery.

## Introduction

Oral and craniofacial tissue repair represents not only a clinically important topic, but also a fascinating system for basic scientific research. The multitude of tissue types in the oral/craniofacial region—including bone, mucosa, tooth, and skin—are defined by distinct developmental origins, cellular properties, and healing outcomes. This complexity presents a clinical challenge, as injury to or congenital defects of the region often involve multiple tissue types, each of which heal differently and may face unique challenges to repair. Across these disparate tissues, however, the desired healing outcome is the same: regeneration.

Regeneration is defined by re-creation of “normal” tissue identical to the endogenous uninjured tissue, rather than injury repair through deposition of fibrotic (scar) tissue, the latter being the typical healing outcome in most adult tissues [1]. While there are some instances of regenerative healing in humans—e.g., scarless skin wound healing in early-gestation fetuses [2]—in general, postnatal human tissues throughout the body exhibit limited or absent regeneration in response to injury [3]. There are many clinical contexts that demand replacement of damaged or missing oral/craniofacial tissue, such as congenital defects, traumatic injury, and head and neck cancer resections. Regeneration in postnatal soft tissues and bone is highly desirable, as therapies or interventions that could produce tissue regeneration would enable restoration of both form and function in diverse clinical contexts.

Interestingly, within the oral/craniofacial region, multiple contrasting healing outcomes—ranging from fibrotic/scarring repair to near-perfect regeneration—exist in concert [4]. These divergent healing modalities within neighboring tissues pose a unique opportunity to study and understand underlying mechanisms of regeneration vs. non-regenerative healing. In this review, we will examine current basic scientific understanding of healing in four tissues relevant to oral and craniofacial surgery, with relevant clinical or preclinical examples drawn from each tissue: (1) Bone (calvarial defects and mandibular distraction osteogenesis); (2) Skin (cleft lip repair); (3) Oral mucosa (intraoral and palatal injury repair); and (4) Tooth (incisor regeneration in rodents). We will discuss conserved and divergent pathways/mechanisms of repair in these different contexts. Ultimately, better understanding of why some oral/craniofacial tissues regenerate while others exhibit fibrotic repair may guide the development of new, targeted molecular and cellular therapies to drive bone and soft tissue regeneration.

## Developmental Origins Of Oral/Craniofacial Tissues

The oral/craniofacial region is in some ways a microcosm of the body: within a relatively small space, it contains a complex conglomeration of many key tissue types, including skin, mucosal epithelium, and bone. However, compared to similar tissues elsewhere in the body, the tissues of the head and neck have unique developmental origins that lend them distinct physiological properties. In some cases, these developmental pathways are recapitulated in the setting of injury repair.

The tissues of the head and neck derive largely from the cranial neural crest, with contributions from the paraxial mesoderm. The neural crest is a specialized group of multipotential, migratory cells that originate from the neuroectoderm [5]. These cells give rise to mesenchymal tissues of the head and neck via epithelial-to-mesenchymal transition (EMT). While these cells are technically derived from the ectoderm, the neural crest is sometimes referred to as the “fourth germ layer,” given its developmental importance [6]. The paraxial (or somitic) mesoderm forms simultaneously with the neural tube and comprises the longitudinal regions of mesodermal tissue on either side of the neural tube [6].

Here, we provide a brief overview of the developmental/embryonic origins of several key craniofacial/oral tissue types, which serve as an important foundation for understanding mechanisms of repair in these tissues.

### Bone

The bony framework of the head comprises 22 bones, most of which become fused together over the course of development (a process that is not fully complete until, typically, 18–24 months after birth [7]). The skull and facial bones are derived from two different embryonic sources. The dorsal part of the skull—including the parietal, occipital, and a portion of the temporal (petrous temporal) bones—is mesodermal in origin, forming from the rostral-most somites of the paraxial mesenchyme [5]. The frontal part of the skull—including the facial, frontal, sphenoid, and squamous temporal bones as well as the bones of the middle ear and jaw—is derived from neural crest ectomesenchyme cells [5, 6]. Neural crest cells also give rise to odontoblasts that will ultimately form part of the dental pulp and produce dentin (a calcified substance that is a major component of teeth; see following section) [6, 8].

Cells of the cranial neural crest, which produce the bones of the face and jaws, are critically regulated by Hox genes. Different Hox genes are expressed along the neural crest cell axis, and specific combinations of Hox genes dictate the fates of these cells [6]. The hindbrain is an important source of this patterning information, with the distinct segments (rhombomeres) of the hindbrain forming restricted expression domains for the different Hox genes [9]. A family of genes called ephrins/Eph receptors are also crucial for hindbrain/neural crest patterning. Alternating classes of ephrins/Eph receptors maintain restricted gene expression domains and prevent mixing during hindbrain development. Eph/ephrin genes also play a key role in regulating neural crest development, by restricting neural crest cell migration [6, 9]. Ultimately, Hedgehog and bone morphogenetic protein (BMP) signaling are critical for bony differentiation of calvarial bones [10–12]. The molecular signals discussed above are important both for specifying bone cell fate (e.g., differentiation to osteoblasts or chondrocytes) and for determining regionally-specific bone properties (e.g., anatomical identities of different skull and face bones). These signals could thus have implications for guiding postnatal regeneration {which can recapitulate molecular signatures of embryonic development [13]} in a tissue type- and regionally-defined manner.

### Teeth

Teeth are complex organs formed from multiple tissue components. Broadly, they comprise an outer, mineralized, hard structure—further subdivided into the outer enamel and underlying dentin—surrounding a soft, living inner tissue, the pulp. The cementum, a third mineralized substance, covers the tooth root and attaches the teeth to the underlying bone via the periodontal ligament, a fibrous structure [14].

These components of teeth are produced by different cell types. Enamel is produced by ameloblasts, a cell type which arises from the oral ectoderm. Dentin is made by odontoblasts; these and the other structures of the tooth develop from neural crest-derived ectomesenchymal cells [15]. Reciprocal, sequential, paracrine epithelial-mesenchymal interactions are critical in informing tooth development [16, 17]. Molecular signaling pathways involved in interactions between the ectodermal and mesenchymal components of the developing tooth include transforming growth factor (TGF), fibroblast growth factor (FGF), Hedgehog, Wnt, and BMP signaling [15, 17]. Interactions also occur between the teeth and the jaws, with transcription factors such as homeobox genes inducing growth factors and patterning of both the set of teeth and the jaws [15]. The fact that the dental complex contains multiple tissues distinguished by different cell types, mechanical properties, and molecular signaling patterns poses a challenge to achieving postnatal regeneration. Any (or multiple) tooth or periodontal components may be deficient in disease states; thus, it is critical to understand the distinct signals that regulate each tissue type in order to most efficiently drive regeneration of the desired tissues.

### Skin

The skin is composed of a keratinized epithelial layer (the epidermis) overlying a mesenchymal layer (dermis). The epidermal layer also gives rise to the skin's appendages, or adnexa, including hair follicles and glands. Epidermis is derived from ectodermal tissue [18]. The *p63* gene is an important regulator of craniofacial epidermal development [19], as are BMPs, which drive ectodermal cells away from a neural fate and toward an epidermal fate [20]. The basal layer of the epidermis also contains melanocytes, specialized pigment-producing cells that arise from the neural crest [20].

Craniofacial dermis arises from two sources: the dermis of the face originates from cranial neural crest cells, while the remaining cranial dermis derives from paraxial (cephalic) mesoderm [18]. Neural crest precursors of dermal cells are guided in their migration by ephrin and semaphorin signaling. Early Wnt signaling is required to commit neural crest cells to a dermal fate [18, 21]. Signaling through multiple pathways, including Wnt, BMP, Sonic hedgehog (Shh), and TGF-B, drives the development of skin appendages through interactions between mesenchymal and epidermal cells, with Wnt signaling being particularly critical for hair follicle development [18, 22]. Skin patterning varies regionally throughout the body (for example, the skin of the scalp is obviously different from the skin of the face). This phenomenon is regulated by Hox gene expression, wherein underlying dermal cells inform patterning of overlying epithelium, and it has been shown that the “positional identity” of dermal fibroblasts is retained past embryonic development [23]. This regional “memory” may have clinical implications, such as distinct therapeutic approaches to target skin regeneration in different regions of the body (particularly between the neural crest-derived facial skin and the mesoderm-derived skin of the rest of the body).

### Oral Mucosa

The oral cavity represents the site of the embryonic communication/junction between ectoderm (defining most of the oral cavity) and endoderm (forming the foregut). The majority of the oral mucosal epithelium (with the exception of the tongue, which forms from endoderm) is ectoderm-derived, while the connective tissue underlying the oral mucosa is formed from the neural crest ectomesenchyme [24]. Neural crest ectomesenchymal cells also form the muscles of the lips, cheeks, and soft palate. Similar to tooth and skin, epithelial-mesenchymal interactions between the mucosal epithelium and underlying mesenchymal tissue are important for patterning and development of these tissues, though the molecular nature of these interactions remains to be precisely characterized.

## Repair And Regeneration Of Craniofacial Bone

### Calvarial Defects

The calvarium (top part of the skull) is made up of multiple bones: the frontal bone, parietal bones, and occipital bone (superior parts of each). At the time of birth, the bones of the skull are not yet fused, allowing for easier passage through the birth canal. Two holes/gaps exist in the newborn skull: the larger anterior fontanelle, and the smaller posterior fontanelle, which can be felt as “soft spots.” There also exist sutures, lines where the different skull bones join together. During normal development, the fontanelles will close by the age of 2 years, while the cranial sutures do not fully fuse until mature adulthood (30–40 years of age). The process of skull development and suture fusion represents a tightly regulated sequence of events, as skull growth must be sufficient to accommodate growth of the developing brain.

Calvarial defects can have many possible etiologies, but all are clinically challenging. As stated above, the process of calvarial development and maturation must be finely coordinated; otherwise, calvarial pathologies can result. Broadly, calvarial development can be dysregulated in two ways. First, premature fusion of the sutures can occur; this pathology, known as craniosynostosis, leads to increased intracranial pressure, which can impair basic brain function. Second, the sutures can fail to fuse appropriately, be enlarged, or be missing sections of bone altogether. In the latter case, these anomalies can directly lead to gaps in the skull which must be repaired. In the former, the only existing treatment for craniosynostosis is surgical release of the pathologically fused suture [25]. While preoperative computer-assisted modeling has aided craniofacial surgeons in planning these procedures so that they can most efficiently remodel the existing skull bones while leaving minimal gaps, inevitably some defects will result where existing bone is insufficient to cover the entire skull vault. Thus, in both of these instances, bony gaps will result, posing a need for regenerative reconstructive measures. In addition, calvarial defects can result from non-congenital sources, e.g., following traumatic injury to the skull or after tumor resection.

The skull has some capacity for endogenous regeneration of defects. However, while young pediatric patients may be able to regenerate sizeable calvarial defects, this ability declines with age [26]. Further, calvarial reconstruction in children faces unique challenges, including the fact that the skull is still growing and developing [27]. Other factors may pose obstacles to endogenous calvarial regeneration, such as prior radiation therapy for treatment of cancer [28]. Current clinical approaches for reconstructing calvarial defects are limited and each suffer from critical drawbacks. One option is autografts (grafting tissue from the patient's own body). In terms of replacing “like with like” tissue and achieving good graft integration, this may be the ideal approach, but obvious drawbacks are donor site morbidity and lack of appropriate available donor tissue for grafting, as well as a chance of graft resorption (which can be upwards of 50% in children) [29–31]. Other options include cadaveric bone allografts, animal xenografts, and synthetic (alloplastic) materials, which may have challenges integrating with the recipient bone, cannot grow with the patient (an issue for pediatric patients), and carry the risk of complications such as infection and extrusion [31, 32]. None of these options are ideal, and complication and failure rates of cranioplasty are high, with the most common complications (such as bone flap resorption) affecting over 80% of young patients and requiring surgical revision in over half of these patients [30, 33, 34]. Thus, there is a need for therapies that can encourage bone regeneration in the skull, which would have widespread and meaningful clinical impact.

Basic science research is currently aimed at developing novel approaches for encouraging regeneration of critical-sized calvarial defects (i.e., those that exceed the body's endogenous capacity for spontaneous repair). First, a large body of work has aimed to identify molecular signaling pathways with the potential to improve calvarial repair. Interestingly, the neural crest-derived bones of the skull exhibit enhanced healing capacity compared to those of mesodermal origin, suggesting that studying the biology of neural crest-derived bones may yield insights into improved skeletal healing [35]. It has been observed that the superior healing ability of neural crest-derived bone involves enhanced Wnt signaling in these bones [35], and that driving supraphysiologic Wnt signaling activation in mesoderm-derived bone via a transgenic mouse model improved the bone's repair capacity [36]. Other studies have looked to the enhanced healing ability of young bones for molecular clues; studies in mice and humans have implicated signaling by osteogenic growth factors such as BMP-2/4/7 and FGF-2 [37, 38]. Encouragingly, mouse, rabbit, and human studies have found that supplementation of FGF-2 and/or BMP-2 improve calvarial bone healing capacity even in older individuals [39–41], a finding with exciting translational applications.

Other studies have examined whether cell-based therapies could offer a viable approach to encourage calvarial defect repair. One group found that human deciduous (“baby”) tooth-derived stem cells delivered to critical-sized mouse calvarial defects resulted in significant bone formation;[42] other studies have applied adult stem cells such as mesenchymal stem cells (MSCs)/adipose-derived stem cells (ASCs) [31]. Combining a cellular and molecular approach, one study reported that by transducing calvarial mesenchymal progenitor cells to express BMP-9 and delivering these cells to critical-sized defects, calvarial healing was improved, with increased osseointegration and mature bone formation [43]. Still other studies have applied a scaffold-based approach, using materials such as extracellular matrix (ECM)-based hydrogels to enhance healing of calvarial defects [44]. While the optimal treatment approach remains to be identified, these findings collectively show promise that manipulating the cellular/molecular environment in bone may facilitate improved healing and regeneration in the setting of calvarial defects.

### Mandibular Distraction Osteogenesis

Distraction osteogenesis (DO) is a surgical procedure used to gradually generate new bone over time. DO is most commonly used in the practice of oral-maxillofacial/craniofacial surgery, where it is applied to repair conditions of bone deficiency such as mandibular hypoplasia (micrognathia), a birth defect wherein the jaw is significantly undersized [45]. In DO, the bone to be lengthened is first cut surgically, and the defect is allowed to heal for a brief period. A device is then affixed across the healing region and gradually expanded over time (~1 mm/day), such that the two pieces of bone are pulled apart (“distracted”) at the site of the injury [46]. The end result is the deposition of new bone tissue across the distracted region (“distraction gap”), which has an overall effect of lengthening/expanding the bone.

It is generally thought that the mechanical stimulus of tension being applied across the injury site is a key driver of the new bone formation observed during distraction [45]. The precise molecular mechanisms of DO are unknown but represent an active area of research, as this unique regenerative process may yield insights into encouraging bone regeneration in other clinically relevant contexts. Numerous studies have reported that classical mechanotransduction signaling [through cell-surface integrins, focal adhesion kinase (FAK), extracellular signal-related kinase (ERK), etc.] are involved in the regenerative response to DO, possibly through upregulating expression of growth factors such as BMP-2/4 [47–49]. Consistent with these *in vivo* findings, mechanical stretching of bone marrow MSCs *in vitro* was found to induce osteogenic differentiation in a FAK-dependent manner [50]. While DO can also be used in long bones of the appendicular skeleton, mandibular DO is of particular biological interest, given the neural crest embryonic origins of the mandible. In fact, the mandible has been reported to heal via neural crest-derived skeletal stem cells (compared to mesoderm-derived stem cells for the tibia) [51]. Most recently, a study linked the mechanical aspects of DO and the mandible's embryonic origins by reporting that mechanically-activated skeletal stem cells in the mandible re-activate neural crest developmental molecular pathways during DO, highlighting a key mechanism for regeneration [13]. Continued study of the mechanisms of DO, particularly in the neural crest-derived bones of the craniofacial skeleton, may yield useful insights into clinically-targetable mechanisms of skeletal regeneration.

## Skin Wound Repair Via Fibrosis/Scarring

### Burden of Facial Scarring: Cleft Lip/Palate and Burns

An estimated 100 million scars are produced each year in the developed world as a result of trauma, burns, and surgical procedures [52]. Worldwide, this burden is estimated to be four- to five-fold higher [52]. Beyond their aesthetic drawbacks, scars substantially compromise skin's form, function, and mechanical robustness. Scarring is particularly detrimental in the craniofacial region, where scars can cause negative psychological effects, carry social stigma, and negatively impact breathing, speech, swallowing, and other essential functions [53, 54].

Wound healing following cleft lip/palate (CL/P) repair is particularly troublesome, with rates of hypertrophic scar formation as high as 36% [55, 56]. The stiff, fibrotic scar tissue resulting from early surgeries (which begin around age 3 months) has lifelong sequelae, as it can restrict craniofacial growth and tissue function and may ultimately lead to speech disorders and nose/jaw deformities [55]. Facial burns also frequently form hypertrophic scars that can cause devastating visual deformation as well as painful and debilitating contractures, particularly in the neck region [57]. Unfortunately, despite decades of research, there are no fully efficacious therapies for minimizing scar formation or promoting wound regeneration in the facial region, highlighting an urgent unmet need [58].

### Mechanisms of Fibrotic and Regenerative Skin Repair

Skin wound healing is classically broken into three overlapping phases following hemostasis: inflammation, proliferation, and maturation/remodeling [3]. In the inflammatory phase, platelet adhesion and activation attracts nearby immune cells and initiates capillary leak to produce the characteristic warmth, swelling, and redness of inflammation [59]. Neutrophils and macrophages infiltrate the wound in the early and late stages of inflammation, respectively, clearing pathogens and debris and elaborating signaling molecules to promote migration of keratinocytes, fibroblasts, and endothelial cells [59, 60]. In the proliferative phase, these cells mediate re-epithelialization of the wound and replacement of the provisional matrix with highly vascularized granulation tissue containing fibronectin, collagen type III, proteoglycans, and other ECM proteins [59, 61]. Finally, during the maturation phase, fibroblasts gradually remodel granulation tissue and replace it with mature scar ECM proteins (e.g., collagen type I), which are later pruned and crosslinked to strengthen the scar [59, 62, 63]. However, the scar ultimately only ever reaches 80% of unwounded skin's original strength [64]. Furthermore, rapid replacement of lost tissue with scar ECM precludes the regeneration of hair follicles, sebaceous glands, and other adnexal structures that confer skin's thermoregulatory, moisture-regulating, and barrier functions, leading to a characteristic “bare area.”

In postnatal life, wounds in facial skin evolve along the above phases and inevitably form scars. However, in 1971, it was shown that incisions in fetal lambs prior to embryonic day 90 (E90) healed with no evidence of scar, complete recovery of adnexa and normal histological architecture, and relatively minimal inflammation [65]. This finding of embryonic scarless wound healing was replicated in a variety of mammalian fetuses and ultimately demonstrated in human fetuses [3, 66]. Regenerative skin repair during early gestation is also observed in facial skin, as full-thickness wounds in fetal rhesus monkey lips prior to E85 heal by complete regeneration [67]. While many differences between the postnatal and *in utero* environments (e.g., growth factors, temperature, oxygen tension, sterility, cell differentiation, immune system maturity) have been investigated to explain the lack of scarring *in utero*, none of these environmental factors appear to be essential [68, 69]. Rather, it is apparent from reciprocal translational experiments that scarless healing is intrinsic to fetal wounds [69, 70].

As the dominant source of scar ECM, fibroblasts have been a major focus of subsequent studies on mechanisms of skin scarring and regeneration. These studies have revealed that fibroblasts are remarkably heterogeneous cells [71]. For example, studies have implicated specific fibroblast lineages in driving scarring, reporting that the vast majority of scarring in the dorsal skin of mice is mediated by *Engrailed-1* lineage-positive fibroblasts [72] while an analogous population of *Prrx1* lineage-positive fibroblasts mediates ventral scarring [73]. Other groups have differentiated fibroblasts by their microanatomical position within the dermis. For example, superficial vs. deeper dermal fibroblasts have been distinguished by their patterns of surface marker expression and transcriptional profiles in both unwounded and wounded skin [74]. As alluded to above, fibroblasts also vary by their embryonic origin, suggesting that intrinsic differences in fibroblasts from craniofacial regions due to their unique neural crest origins may influence their scarring behavior [23]. Further work is needed to reconcile the various “lenses” of fibroblast heterogeneity in order to identify conserved, common master regulators of scarring and regenerative function [71]. In addition, studies should determine whether the neural crest origin specific to facial dermal fibroblasts lends them distinct cellular/molecular properties. Epigenomic comparison of fetal regenerative (prior to E16.5 in mice) and scarring (after E18) fibroblasts may also reveal differential regulation of such master regulators, which would represent attractive therapeutic targets to promote craniofacial wound regeneration in postnatal life.

## Mucosal Repair In the Oral Cavity

### Minimally Scarring Oral Healing

The oral mucosa is generally similar in structure to skin, with a stratified epithelium overlying a dermis, and heals via the same phases as skin. However, while skin wounds heal by scarring, the oral mucosa rapidly heals by regeneration with little to no apparent scar [75]. These differences in healing outcomes may be evolutionarily advantageous. The oral cavity experiences frequent trauma during eating and would be rendered dysfunctional by scarring; in contrast, the skin experiences less-frequent trauma and, from an evolutionary perspective, requires rapid replacement of lost tissue with scar to prevent infection. Oral wounds re-epithelialize more rapidly than skin wounds [76], exhibit reduced inflammation [2, 77], and heal with fibronectin-rich ECM, much like fetal wounds [2, 78]. Furthermore, oral wounds are more vascular than postnatal skin wounds [78] and show a decreased ratio of matrix metalloproteinases (MMPs) to tissue inhibitors of metalloproteinases (TIMPs), indicating higher levels of active ECM remodeling [79].

Several extrinsic differences between the oral cavity and skin may explain their differences in healing outcomes. Oral mucosa is continuously bathed in saliva, which contains epidermal growth factor (EGF), which accelerates re-epithelialization, and FGF, which acts on fibroblasts to increase their turnover and promote wound closure [80–82]. Saliva also contains histatins, hydrogen peroxide, lactoferrin, and lysozymes that provide crucial antimicrobial defense [82]. In response to injury, oral mucosa exhibits faster resolution of inflammation and generally lower levels of inflammatory cytokines, similar to fetal skin [77, 83–85]. Interestingly, oral epithelial cells also show a muted response to inflammatory cytokines compared to skin epithelial cells, suggesting that the oral mucosa is intrinsically less sensitive to inflammation [86]. Oral epithelial cells may also be less prone to differentiation than skin epithelial cells, instead preferentially adopting a proliferative and migratory phenotype [77, 78]. Transcriptional profiling of oral mucosal epithelium has revealed relatively fewer gene changes upon wounding and high baseline expression of genes related to repair pathways, suggesting an intrinsic “readiness” to respond to injury [77, 86]. Several other studies have revealed intrinsic differences between oral mucosal and skin fibroblasts. *Wnt1* lineage-positive fibroblasts of the oral mucosa are non-scarring even when transplanted to dorsal skin, whereas dorsal skin *Engrailed-1* lineage-positive fibroblasts retain pro-fibrogenic behavior when transplanted into oral mucosa [72]. This finding suggests that oral mucosal fibroblasts are intrinsically less fibrogenic than skin fibroblasts. Other studies have revealed that oral cavity fibroblasts replicate at a faster rate than skin fibroblasts and exhibit reduced differentiation into myofibroblasts [87], consistent with their lower propensity for scar formation.

### Problematic Oral Healing

Despite its regenerative nature, oral mucosal healing may be compromised in certain clinical contexts. For example, oral mucosal wounds may fail to heal in the setting of infection, nerve damage, steroid use, diabetic microangiopathy, cancer, nutritional problems, or foreign body presence, much like skin wounds [57, 75, 88]. Cigarette smoking is also associated with impaired oral healing [89]. Interestingly, severe palatal fibrosis can occur in the absence of healthy underlying bone, suggesting important crosstalk between the oral mucosa and surrounding connective tissues [88]. Failure of the oral mucosa to heal may manifest as a malodorous, exudative, dehisced, or necrotic wound [57, 75, 88]. In the context of CL/P repair, poor wound healing can result in oronasal fistula and nasal regurgitation of liquid/food [90, 91]. Poor oral mucosal healing can also directly result from scarring following primary CL/P repair, as the fibrotic skin/palatal mucosal scar impedes blood flow and tethers the maxilla, restricting its growth; failure of the maxilla to extend laterally and anteriorly in turn causes velopharyngeal dysfunction, requiring additional corrective surgeries [91–93]. It is estimated that maxillary hypoplasia secondary to skin/palatal mucosal scarring occurs after up to 50% of CL/P repairs [93], incurring additional procedures with attendant anesthetic burden and cost.

While poorly-healing oral wounds lack a definitive treatment, several avenues for improving impaired oral healing are currently being explored. These strategies—which include electrical stimulation of tissue, photobiomodulation therapy, and growth factor treatments—have shown promise for improving clinical healing of chronic skin wounds [94–96] but have been less explored for oral healing. Current research seeks to determine whether these therapeutic modalities can also be useful for problematic intraoral wound healing. For instance, rodent studies suggest that electrical stimulation may also be useful for supporting intraoral (e.g., palatal, gingival) wound healing [94, 97]. Photobiomodulation therapy has also been shown to improve healing in patients following palatal graft harvest [98]. Finally, treatment with platelet-rich fibrin (a source of concentrated growth factors, platelets, and wound healing cell types such as white blood cells) has been shown by multiple studies to support soft tissue healing/regeneration (e.g., treating gingival recession) [99]. Given the high prevalence of conditions that predispose individuals to impaired oral healing (e.g., cigarette smoking, diabetes), it will be important to identify optimal therapeutic approaches for supporting oral healing and regeneration in clinically relevant settings.

## Approaches For Tooth Regeneration

### Enormous Burden of Oral/Dental Disease

Oral diseases, including dental caries and periodontal disease, are some of the most prevalent diseases throughout the world. Dental caries (“cavities”) has been reported as the most common disease process in the U.S., with over half of all adolescents (12–19 years old) and roughly 90% of adults (20 years or older) experiencing dental caries [100]. Periodontal disease (“gum disease”), which is estimated to affect 20–50% of the worldwide population, damages the tissues surrounding the teeth and, over time, can lead to destruction of tooth attachments and underlying alveolar bone; as such, it is the leading cause of tooth loss [101]. Globally, as of 2015, 3.5 *billion* people suffered from untreated dental caries, periodontal disease, and/or edentulism (tooth loss) [102]. Tooth disease can cause problems with eating/chewing, social interaction, self-esteem, and pain [103]. Untreated dental disease disproportionately affects individuals who are older, of lower socioeconomic status, and/or racial/ethnic minorities [103, 104].

### Periodontal Regeneration Approaches

Given the substantial burden of periodontal disease and its sequelae (most notably, tooth loss), regeneration of diseased periodontal tissues has long been an attractive therapeutic target. However, current clinical treatments are largely limited to minimizing disease progression and managing symptoms (e.g., pain) [105]. Ideally, regenerative therapies for periodontal disease would induce regeneration of all damaged components of the periodontal tissues, including the alveolar bone, periodontal ligament, and cementum, and would integrate with the tooth root to restore a functional and robust tooth attachment. Theoretically, conventional treatments—which include surgical debridement/resection of diseased tissues—can result in regeneration of bone and supporting structures; in reality, occurrence of such spontaneous regeneration is rare and limited to isolated clinical reports [106]. However, these outcomes highlight the fact that cells intrinsically capable of postnatal regeneration of periodontal tissues do exist within the native periodontal niche. Thus, work toward novel regenerative therapies has largely focused on supporting and encouraging regenerative activity of these native cells.

#### Guided Tissue Regeneration

One broad clinical strategy for periodontal regeneration has been guided tissue regeneration (GTR) following debridement of diseased tissue. This approach involves inserting a barrier membrane between the deeper periodontal tissues (where regeneration-competent cells are presumed to reside) and the more superficial gingival tissue, in order to prevent epithelial downgrowth from the upper compartment and enable pro-regenerative periodontal ligamentous cells to enter the defect site [106, 107]. While different GTR approaches have been actively studied in recent decades—with a large body of literature dedicated to different materials [108], e.g., resorbable vs. non-resorbable membrane options—substantial heterogeneity exists between the conclusions and degree of benefit supported by these studies [105, 109]. In addition, GTR is not without its own hazards; for instance, non-resorbable membranes, while shown to improve regenerative outcomes compared to resorbable membranes, are associated with relatively high complication rates (e.g., infection) and require a secondary procedure to remove the membrane following treatment [105, 109].

#### Periodontal Tissue Engineering

A second therapeutic direction has been the development of tissue engineering approaches for periodontal regeneration. An extensive body of work has explored specific periodontal tissue engineering approaches with different biomaterials, molecular factors, and synthesis methods. While an in-depth discussion of these strategies is outside of the scope of this review, we will highlight some general principles of the most promising regenerative approaches. First, given the complex milieu of cell types within the periodontal complex, scaffold-based (rather than cell-based) approaches are favorable [105] {though stem cell transplantation approaches remain another active area of research [110, 111]}. Similarly, as the periodontal tissue complex involves multiple tissue types (bone, cementum, and soft tissue ligament) with distinct physical properties, composite biomaterials—for instance, ones containing multiple layers that recapitulate features of the different periodontal tissue types—are needed for full regeneration [105]. As our ability to control biomaterials production advances to smaller and smaller scales, the ability to produce increasingly biomimetic and micro-patterned scaffolds continues to improve [112]. Researchers are also interested in incorporation of molecular/growth factors to stimulate cell differentiation into desired lineages. For example, supplementation with BMPs (to support chondroblast/osteoblast formation), synthetic cell-binding peptides (to support homing/infiltration of cells into the scaffold [113]), or FGFs (to support recruitment and proliferation of soft tissue cells such as endothelial and periodontal ligament cells) represent avenues of research [114]. However, it is important to note that most of these strategies remain far from widespread clinical application, and patient studies will ultimately be needed to determine whether these treatments result in consistent regenerative benefits.

### Challenges for Tooth Regeneration

Unfortunately, teeth have a very limited capacity for self-repair [14]. Treatments for dental disease (e.g., caries) generally involve removal of diseased/decayed tissue and use of synthetic/inert restorative materials to re-establish normal tooth structure and restore function [115]. Research has led to substantial improvements in the quality and longevity of dental repair materials over time. However, as these materials are non-bioactive, they cannot fully integrate with or regenerate the native living tooth tissue. As such, repair failure is a known and unavoidable risk, albeit an increasingly rare one {with annual failure rates for modern composites ranging from 1 to 5% per year [116]}. In addition, for more severe disease such as complete tooth loss, treatment options are limited. There is no way to replace a lost tooth with a new, living tooth; instead, patients must live with bridges/dentures (which have many drawbacks, including lacking the appearance of native teeth, discomfort, and challenges with eating and speaking) or tooth implants (which are expensive and typically not covered by insurance, making them inaccessible to most patients) [117]. In addition to often prohibitive costs, tooth implants involve attaching inert materials directly to the underlying bone (in contrast to the native structure, where the tooth is anchored to bone via an intervening periodontal ligament); this could lead to imbalanced translation of masticatory forces to the jaw, ultimately predisposing to jaw bone resorption [118]. Ideally, therapies would be developed that would allow for tooth regeneration—either inducing native teeth to regenerate injured/decayed tissue, or creating methods to regenerate tooth tissue outside of the body that could then be transplanted for therapeutic use.

#### Targeting Dental Stem Cell Signaling

Multipotent stem cells exist in the dental pulp that are believed to be capable of regenerating dentin and pulp-like tissue [119, 120]. However, the reparative ability of these dental pulp stem cells in human teeth is limited, and they can only mobilize to repair injuries up to a critical size [118]. Researchers have sought to identify strategies to improve on the repair potential of these existing stem cells within the teeth via manipulating molecular signaling pathways. Wnt signaling seems to be a promising target. When collagen sponges containing small molecule inhibitors of glycogen synthase kinase (GSK-3, a Wnt antagonist) were placed into mouse tooth injury sites, mineralization was increased, with native-like dentin deposited at the injury site to replace the sponge as it degraded over time [121, 122]. Another study showed that the type 2 diabetes drug metformin increases odontoblastic differentiation of dental pulp cells *in vitro* by activating the adenosine monophosphate-activated protein kinase (AMPK) pathway [123]. This group was able to incorporate metformin into a dental resin with similar effects on dental pulp cells [124], suggesting a possible direct translational pathway via treating tooth defects/caries with drug-containing rather than traditional inert resins.

#### Mouse Incisors as a Model of Tooth Regeneration

Other studies have looked to animal models for clues to tooth regeneration. Rodent incisors grow continually throughout the lifetime of the animal, presenting a model of endogenous tooth regeneration in an adult mammal. One study found that signaling through FGF receptor 2b was necessary for normal incisor stem cell development as well as adult incisor regeneration [125]. Another study further characterized the stem cell dynamics of the mouse incisor using single-cell RNA-sequencing and identified that actively cycling epithelial progenitors contribute to incisor growth during homeostasis; injury repair involved both increased proliferation of these progenitor cells and conversion of Notch1-expressing cells under the enamel directly into enamel-producing ameloblasts [126]. Notch and Delta-like 1 homolog (Dlk1; a non-canonical Notch ligand) have also been implicated in regulating the homeostatic balance between stem/progenitor cell populations in the mouse incisor [127].

#### Tissue Engineering and Cell-Based Therapies for Tooth Regeneration

A relatively large body of work has examined the possibility of using cell-based therapies for tooth regeneration. The human tooth contains multiple stem and progenitor cell populations/reservoirs [14, 128]; these can be obtained from many readily available sources (e.g., extracted third molars/wisdom teeth, exfoliated deciduous teeth) [129]. Tooth-resident stem cells may hold promise for regeneration, as they exhibit differentiation potential for key dental cell types. In addition, the ability to potentially derive these cells from a patient's own exfoliated or removed teeth raises the possibility of banking a patient's dental stem cells, such that patient-specific therapies could be derived if the need arose [128]. Researchers have also developed other strategies for generating stem cells capable of differentiating into dental cell types, such as via differentiation of embryonic or induced pluripotent stem cells (ESCs/iPSCs) [130]. Substantial interest exists in approaches that may be able to generate engineered whole teeth to replace those that have been lost or extensively damaged. New developments in biomaterials (to provide scaffolds for growing tooth tissue *ex vivo*) and the understanding and culture of dental stem cells may hold the key to growing teeth in the lab [131]. It must be noted that whole tooth regeneration remains far from clinical implementation, and significant work is still needed to demonstrate both feasibility and utility of such tissue engineering approaches. However, the ability to replace dental tissue or even whole teeth with bioactive materials capable of integrating into the native dental/oral milieu would represent a significant advancement from current treatment options.

## Conclusion

The complexity in structure, cellular composition, molecular signaling, and developmental origins of the craniofacial tissues is reflected in their diversity of healing outcomes and regenerative capacity. For example, while injuries to the oral mucosal epithelium and underlying mesenchyme heal in a minimally scarring fashion, injuries to the external facial skin invariably heal by forming fibrotic scars. Craniofacial bone and teeth exhibit some endogenous capacity for repair/regeneration, but this regenerative ability is limited, and instances of critical defects (which exceed the body's intrinsic ability to repair the injury) represent widespread and significant clinical issues. Throughout the craniofacial region, regeneration of normal tissue is a highly desirable clinical goal. While this goal remains elusive, basic science research continues to yield important discoveries into mechanisms of repair and regeneration. In this review, we highlight key clinical problems in four key craniofacial tissues—bone, skin, mucosa, and tooth—where the fundamental detriment results from lack of regeneration. We also survey current scientific knowledge of the mechanisms, molecular drivers, and potential translational directions for encouraging improved regeneration in each of these tissues. Elucidating precise mechanisms of regenerative vs. non-regenerative repair in craniofacial tissues may form the foundation for the development of new, pro-regenerative therapeutic targets and strategies.

## Author Contributions

HEdJ-P and SM researched and wrote the manuscript. MTL and DCW edited the manuscript. All authors contributed to the article and approved the submitted version.

## Conflict of Interest

The authors declare that the research was conducted in the absence of any commercial or financial relationships that could be construed as a potential conflict of interest.
